# The ATF4-glutamine axis: a central node in cancer metabolism, stress adaptation, and therapeutic targeting

**DOI:** 10.1038/s41420-025-02683-7

**Published:** 2025-08-19

**Authors:** Xing Yan, Changhong Liu

**Affiliations:** The Second Affiliated Hospital of Dalian Medical University Thoracic surgery, DaLian, Liaoning PR China

**Keywords:** Cancer metabolism, Apoptosis

## Abstract

At the center of tumor(neoplasm) metabolic adaptation lies activating transcription factor 4 (ATF4), a key regulator that orchestrates Glutamine (Gln) uptake, utilization, and redox balance under conditions of nutrient deprivation and oxidative stress. This review explores how ATF4 integrates environmental and cellular stress signals to drive Gln metabolic processes, enabling tumor survival, metabolic reprogramming, and immune evasion. The ATF4-Gln axis emerges as a pivotal vulnerability in cancer metabolic processes. Preclinical studies of small-molecule inhibitors and synthetic derivatives disrupting this pathway show promising results. Understanding the intricate interplay between ATF4, Gln metabolic processes, and cancer progression provides valuable insights for novel therapeutic strategies. Future research must address tumor heterogeneity and metabolic flexibility to fully harness the potential of ATF4-centered therapies. However, challenges such as off-target effects of ATF4 inhibitors and metabolic plasticity in tumors remain critical barriers. Future studies integrating multi-omics approaches and AI-driven drug discovery are warranted to overcome these hurdles.

## Facts

- ATF4 is a core transcription factor of the Integrated Stress Response (ISR). Under hypoxic, nutrient-deprived, and oxidative stress conditions in the tumor microenvironment, ATF4 is persistently upregulated, enhancing the metabolic plasticity of tumor cells by regulating genes associated with glutamine (Gln) metabolism, such as the transporter SLC1A5/ASCT2 and the enzyme GLS.
- The ATF4-glutamine axis drives tumor metabolic reprogramming by promoting glutamine uptake, anaplerosis of the TCA cycle, nucleotide synthesis, and production of the antioxidant glutathione (GSH), thereby supporting tumor growth and immune evasion.
- ATF4 exhibits dual functions in tumor progression: its hyperactivation promotes tumor survival, whereas under sustained stress, it can also induce apoptosis through the CHOP pathway. Although drugs targeting ATF4-Gln axis (e.g., DHA [dihydroartemisinin] inducing ATF4 degradation, SLC1A5 monoclonal antibodies) show efficacy in preclinical studies, they face challenges including metabolic plasticity, tumor heterogeneity, and toxicity to normal tissues.

## Open Questions

- How does the heterogeneity of ATF4 expression across tumor types (e.g., breast cancer, pancreatic cancer) and developmental stages influence its interplay with glutamine metabolism?
- Does the metabolic crosstalk of glutamine between tumor cells and stromal cells (e.g., CAFs) modulate ATF4 activity? Furthermore, could targeting this axis reprogram stroma-dependent immunosuppression in the tumor microenvironment?
- Can multi-omics approaches—such as spatial transcriptomics coupled with metabolomics—resolve the spatiotemporal dynamics of the ATF4-glutamine axis? How might AI models predict patient subpopulations with optimal therapeutic response to metabolic inhibitors?
- Furthermore, would targeting epigenetic regulators of ATF4 (e.g., modifications of upstream open reading frames [uORFs] or histone marks) demonstrate superior selectivity compared to direct inhibition of metabolic enzymes like GLS?

## Introduction

Cancer represents a significant global health challenge, characterized by uncontrolled cell proliferation, metabolic dysregulation, and exceptional adaptability to environmental stressors [1–3]. Among these adaptations, metabolic reprogramming plays a central role in enabling cancer cells to survive and thrive under harsh microenvironmental conditions by fulfilling energy demands, maintaining intracellular homeostasis, and driving biosynthetic processes (Tumor acidosis: from the passenger to the driver’s seat; ATF4-Induced Metabolic Reprograming Is a Synthetic Vulnerability of the p62-Deficient Tumor Stroma). Among various metabolic pathways, glutamine (Gln) metabolism occupies a pivotal position. Glutamine serves not only as a vital carbon and nitrogen source for rapidly proliferating tumor cells, but also contributes to redox balance maintenance, antioxidant synthesis (such as glutathione), and supports the TCA cycle and energy production (Elucidation of an mTORC2-PKC-NRF2 pathway that sustains the ATF4 stress response and identification of Sirt5 as a key ATF4 effector). Therefore, Gln metabolism is often referred to as the “fuel pipeline” of tumor metabolism (Reprogramming of glutamine metabolism and its impact on immune response in the tumor microenvironment).

Importantly, the ATF4-glutamine metabolic axis acts as a critical node integrating metabolic and stress signaling, playing an essential role in cancer biology (Novel functions of ATF4 in early stages of pancreatic cancer tumorigenesis). Activating Transcription Factor 4 (ATF4), a key transcriptional regulator in the Integrated Stress Response (ISR), is persistently upregulated in multiple cancer types and further induced under tumor microenvironmental stressors such as hypoxia, nutrient deprivation, and oxidative stress (Hypoxia-Mediated ATF4 Induction Promotes Survival in Detached Conditions in Metastatic Murine Mammary Cancer Cells) [2]. ATF4 activates a broad transcriptional program that regulates amino acid metabolism, antioxidant defense, protein folding, and autophagy, thereby conferring significant metabolic plasticity and survival advantages to cancer cells (Transcriptional and metabolic remodeling in clear cell renal cell carcinoma caused by ATF4 activation and the integrated stress response (ISR)). Specifically, ATF4 promotes glutamine uptake and utilization by upregulating glutamine transporters (e.g., SLC1A5/ASCT2) and metabolic enzymes (e.g., GLS), enhancing glutamine dependency under metabolic stress (The GLS1 inhibitor IPN60090 enhances antitumor immune response through metabolic reprogramming of T cells and impacts on the tumor microenvironment). Conversely, glutamine deprivation can induce ATF4 expression, forming a feedback loop that further enhances tumor adaptability to fluctuating nutrient conditions (Uncovering crosstalk between AMPK and the ATF4-Integrated Stress Response). This bidirectional regulatory relationship forms the foundation of the ATF4-Gln axis—a key mechanism by which cancer cells adapt to microenvironmental stress (Hypoxia-mediated ATF4 induction promotes survival in detached conditions in metastatic murine mammary cancer cells).

Recent studies have unveiled novel mechanisms by which ATF4 regulates glutamine metabolism across various cancer contexts [3]. Beyond its classical role in transcriptional regulation of metabolic enzymes, ATF4 has been implicated in immune modulation and remodeling of the tumor microenvironment (Osteoclast-Cancer Cell Metabolic Crosstalk Confers PARP Inhibitor Resistance in Bone Metastatic Breast Cancer; Metabolic reprogramming by GCN1 mediates metastatic reactivation and outgrowth at liver metastatic site in response to glutamine deprivation in pancreatic cancer). For instance, ATF4-driven glutathione biosynthesis enhances the capacity of cancer cells to scavenge reactive oxygen species (ROS), thereby promoting redox homeostasis (Activation of LXR β inhibits tumor respiration and is synthetically lethal with Bcl-xL inhibition). Moreover, ATF4-mediated upregulation of amino acid transporters facilitates increased nutrient uptake under chemotherapeutic stress (Activated amino acid response pathway generates apatinib resistance by reprograming glutamine metabolism in non-small-cell lung cancer). It also governs the expression of autophagy-related genes, supporting cell survival under therapy-induced stress conditions. Notably, the functional role of ATF4 is highly context-dependent and exhibits a dualistic nature—it can act as an oncogene in certain settings while demonstrating tumor-suppressive properties in others (Shared gene regulatory strategies for the p53 and ATF4-dependent transcriptional networks). This dichotomy highlights the necessity of delineating its context-specific functions, particularly the spatiotemporal dynamics by which it integrates with glutamine metabolic pathways under varying genetic and microenvironmental conditions (Deciphering the role of integrated stress response (ISR) in the developmental stages of mutant KRAS lung cancer; Metabolic signature and response to glutamine deprivation is independent of p53 status in B cell malignancies). Beyond metabolic adaptation, the ATF4-glutamine axis contributes to several hallmarks of cancer, including resistance to cell death, enhanced metastatic potential, and immune evasion. Emerging evidence further implicates this axis in modulating responses to immunotherapy and contributing to treatment resistance, although its underlying molecular mechanisms remain to be fully elucidated (Acute Immune Signatures and Their Legacies in Severe Acute Respiratory Syndrome Coronavirus-2 Infected Cancer Patients).

In summary, the ATF4-glutamine metabolic axis serves as a critical link between cancer cell metabolic demands and adaptive stress responses, thereby influencing both therapeutic outcomes and tumor progression (Mitochondrial pyruvate import is a metabolic vulnerability in androgen receptor-driven prostate cancer). This review synthesizes recent advances to present a comprehensive framework that underscores the central role of this axis in tumor metabolic regulation and its promise as a therapeutic target. Future research should aim to unravel how ATF4 activity is dynamically regulated by glutamine flux and how this interplay affects treatment responsiveness across diverse cancer types, ultimately paving the way for more effective, personalized therapeutic strategies.

## Structure and expression regulation of ATF4

Activating transcription factor 4 (ATF4), also known as CREB-2, is a member of the activating transcription factor/cyclic AMP response element-binding protein (ATF/CREB) family. Structurally, ATF4 comprises a highly conserved basic leucine zipper (bZIP) domain, a transcriptional activation domain, regulatory elements, and a flexible linker region.

**Fig. 1:**
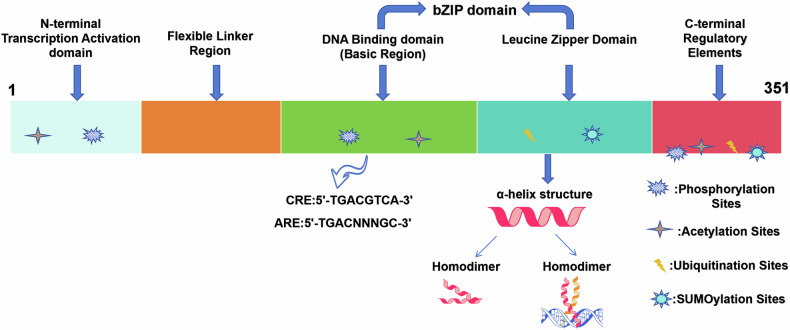
Illustrates the domains and features of ATF4.

The structural organization of ATF4 underlies its functional versatility in stress response regulation. The bZIP domain facilitates the binding of ATF4 to specific DNA sequences in the promoters of target genes, such as cyclic AMP response elements (CREs) and amino acid response elements (AAREs). Furthermore, it enables dimer formation through coiled-coil interactions with other bZIP transcription factors, including CHOP (C/EBP homologous protein), Nrf2 (nuclear factor E2-related factor 2), and c-Myc [4]. Dimerization enhances DNA-binding stability and target gene specificity, thus amplifying ATF4’s transcriptional regulatory capacity. Adjacent to the bZIP region, the transcriptional activation domain located in the N-terminal region recruits co-activators or forms functional heterodimers with other transcription machinery to initiate or amplify gene expression [5] (Fig. 1).

Regulatory elements in the 5′-untranslated region (5′-UTR) include two upstream open reading frames (uORF1 and uORF2). Under normal conditions, uORF1 facilitates ribosomal scanning to uORF2, which inhibits ATF4 translation by causing ribosome stalling. Under stress, however, phosphorylation of eIF2α redirects ribosomes to bypass uORF2, allowing translation of the ATF4 coding sequence [6].

The flexible linker region provides conformational adaptability, enabling ATF4 to interact with various co-factors and adapt to dynamic transcriptional demands [7]. Together, these structural domains define ATF4’s role as a pivotal modulator of cellular adaptation. It activates target genes involved in amino acid metabolism, antioxidant defense, and stress-induced homeostasis.

Figure 1 illustrates the domains and features of ATF4. The transcriptional activation domain is located at the N-terminus of ATF4, where it activates the transcription of target genes by recruiting RNA polymerase II. The regulatory region contains several phosphorylation sites and other potential protein modification sites, allowing it to sense cellular stress conditions. The basic DNA-binding domain recognizes and binds to specific DNA sequences in the promoters of target genes, such as the cAMP Response Element (CRE: 5′-TGACGTCA-3′) or the Antioxidant Response Element (ARE: 5′-TGACnnnGC-3′, where “n” represents any nucleotide). The leucine zipper domain, characterized by repeated leucine residues spaced every seventh amino acid, forms a stable α-helix. This domain enables ATF4 to bind to its own leucine zipper region to form a stable homodimer or to interact with other bZIP family members to form heterodimers, such as ATF3, ATF5, CHOP, or MafK.

To ensure proper stress responsiveness, ATF4 expression is under multilayered control. It is tightly regulated to maintain low basal activity under physiological conditions, while rapidly upregulated in response to stress. This regulation primarily occurs at the translational and post-translational levels, though transcriptional control also plays a role in certain contexts.

### Transcriptional regulation

ATF4 transcription is modulated by promoter elements, epigenetic modifications, and non-coding RNAs (ncRNAs). The ATF4 promoter contains multiple stress response elements, such as antioxidant response elements (AREs) and endoplasmic reticulum stress response elements (ERSEs) [8]. These sites recruit stress-induced transcription factors. For example, under oxidative stress, Nrf2 binds to AREs, while during ER stress, ATF6 and XBP1 engage ERSEs to upregulate ATF4 [9]. Under basal conditions, inhibitory transcription factors like MafK antagonize activators at AP-1-like sites, inducing a closed chromatin configuration that impedes transcription [10, 11].

Epigenetic regulation also shapes ATF4 transcription. DNA methylation within the promoter’s CpG islands blocks transcription factor binding and attracts repressive complexes like MeCP2 and HDACs [12, 13]. Conversely, promoter demethylation enhances chromatin accessibility and gene activation [14]. Histone acetylation (e.g., H3K27ac by CBP/p300) further loosens chromatin, facilitating transcription [15]. Depending on the methylation site and degree, histone methylation may either activate or repress transcription [16, 17], ncRNA add a layer of post-transcriptional control. For instance, miR-200c targets the 3′-UTR of ATF4 mRNA, leading to degradation via the RNA-induced silencing complex (RISC), thereby suppressing translation [18]. These coordinated transcriptional mechanisms ensure that ATF4 is selectively expressed in response to specific stress cues.

### Translational regulation and the ISR

The most dynamic regulation of ATF4 occurs at the level of translation, particularly through the integrated stress response (ISR). Central to this process is eIF2α phosphorylation, which reprograms ribosomal scanning to favor ATF4 translation. Stress-activated kinases—GCN2 (amino acid deprivation) [19], PERK (ER stress) [20], PKR (viral dsRNA) [21], and HRI (heme deficiency) [22]—phosphorylate eIF2α at Ser51 [23]. This halts global protein synthesis but facilitates selective translation of ATF4 by allowing ribosomes to bypass inhibitory uORF2.Such selective translation equips cells to prioritize adaptive proteins without exhausting energy resources, a key goal of the ISR [24].

### Post-translational modifications

After synthesis, ATF4 undergoes rapid turnover and modification, mainly through ubiquitination. This ensures tight temporal control of its activity. Phosphorylation by kinases like GSK-3β at serine 219 primes ATF4 for recognition by β-TrCP, an E3 ubiquitin ligase. This leads to K48- or K63-linked polyubiquitin chain attachment and degradation via the 26S proteasome [25–30]. Under normal conditions, this process prevents ATF4 overaccumulation, maintaining cellular homeostasis. In contrast, during stress, ubiquitination may be attenuated, allowing ATF4 to accumulate and mediate adaptive transcriptional responses.

## The role of ATF4 in cancer

### Metabolic reprogramming regulation

#### Effect on glucose metabolic processes

Tumor cells demonstrate an exceptionally high demand for glucose, frequently surpassing the available extracellular resources. To cope with this metabolic challenge, ATF4 plays a critical role in enhancing glucose utilization by upregulating key components of the glycolytic pathway. In breast cancer [31] and lung cancer [32] cells, ATF4 binds to cis-regulatory elements within the promoter region of the glucose transporter 1 (GLUT1) gene, significantly activating its transcription. The resulting increase in GLUT1 expression facilitates rapid glucose uptake into cancer cells, meeting the heightened metabolic requirements of accelerated proliferation. This regulatory mechanism directly supports the “Warburg effect,” a hallmark of cancer metabolic processes characterized by a preferential reliance on aerobic glycolysis over oxidative phosphorylation for energy production [33]. In addition to GLUT1, ATF4 upregulates the expression of essential glycolytic enzymes, such as 6-phosphofructo-2-kinase/fructose-2,6-bisphosphatase 3 (PFKFB3) [34] and hexokinase 2 (HK2) [35]. PFKFB3 enhances glycolysis by increasing intracellular fructose-2,6-bisphosphate levels, while HK2, the first rate-limiting enzyme in glycolysis, drives glucose phosphorylation, further boosting the glycolytic flux. Moreover, ATF4 promotes the expression of lactate dehydrogenase A (LDHA), which catalyzes the conversion of pyruvate to lactate, regenerating NAD^+^ to sustain glycolytic activity [36]. This ATF4-mediated upregulation of LDHA leads to lactate accumulation in the tumor microenvironment, causing acidification that suppresses immune cell activity and facilitates immune evasion. These ATF4-driven metabolic adaptations not only provide energy and biosynthetic precursors necessary for tumor growth but also enable cancer cells to survive and proliferate under metabolically challenging conditions. By supporting the glycolytic shift, ATF4 enhances malignancy, promotes immune evasion, and contributes to therapeutic resistance, making it a central player in the metabolic reprogramming of cancer cells.

#### Regulation of fatty acid metabolic processes

During the rapid proliferation of tumor cells, large amounts of fatty acids are required to synthesize new cell membranes. Most cancers enhance glycolysis to support fatty acid (FA) synthesis and proliferation under non-stress conditions [37]. ATF4 plays a pivotal role in promoting FA metabolic processes, establishing the necessary metabolic conditions for tumor growth and proliferation [38]. Notably, the inhibition of fatty acid synthase (FASN), a key enzyme in de novo lipogenesis, has been shown to trigger endoplasmic reticulum (ER) stress in tumor cells [39]. In an ovarian cancer cell model, Yang et al. [40] demonstrated that inhibiting FASN leads to the transcriptional upregulation of REDD1, mediated by the ER stress transcription factor ATF4, which prevents de novo FA synthesis and ultimately reduces tumor cell survival. In addition to FASN, ATF4 regulates other critical enzymes involved in FA synthesis. Ren et al. [41] reported that ATF4 directly upregulates the expression of acetyl-CoA carboxylase (ACC), an enzyme that catalyzes the conversion of acetyl-CoA to malonyl-CoA, a rate-limiting step in FA synthesis. Their RT-qPCR results revealed that ATF4 overexpression significantly increased the protein levels of ACC and FASN (*P* < 0.05), whereas ATF4 deficiency led to a significant reduction in the mRNA levels of upstream transcription factors ChREBP and SREBP-1c, as well as ACC and FASN (P < 0.05, *P* < 0.01). Chen et al. [42] further highlighted that ATF4 stabilizes the SREBP1c protein by directly activating the expression of USP7 during adipocyte differentiation, thereby reducing FA synthesis. Beyond individual enzymes, ATF4 contributes to broader transcriptional regulation. Chen et al. [43] demonstrated, through RNA sequencing, that ATF4 and CTCF (a zinc finger protein) form a nuclear complex and co-localize at the promoters of multiple FA-related genes, including C/EBPδ and PPARγ. This complex activates and regulates downstream lipogenic factors, such as fatty acid-binding protein 4 (FABP4), FASN, and ACC, enhancing the ability of tumor cells to synthesize fatty acids. These findings underscore the central role of ATF4 in regulating FA metabolic processes and its importance in supporting the aggressive proliferation of cancer cells.

#### Amino acid metabolism regulation

Amino acids serve as vital nutrient sources for tumor cells, playing an indispensable role in sustaining their rapid growth and survival. ATF4 acts as a central regulator of amino acid homeostasis in the tumor microenvironment, operating through both direct and indirect mechanisms. Under conditions of amino acid abundance, upstream open reading frames (uORFs) in the 5′-UTR of ATF4 mRNA interact with ribosomes to suppress translation of the core coding region, maintaining low basal levels of ATF4. However, during amino acid depletion, the integrated stress response (ISR) is activated. Accumulated uncharged tRNA molecules bind to GCN2, serving as sensors of amino acid scarcity. This interaction triggers the phosphorylation of eIF2α, selectively enhancing ATF4 translation. Subsequently, ATF4 transcriptionally upregulates a variety of genes encoding amino acid transporters and synthases, thereby increasing amino acid acquisition [44]. Numerous studies have revealed a direct connection between oncogenic signaling and the adaptive amino acid response pathway. Gwinn et al. [45] demonstrated that in KRAS-mutated non-small cell lung cancer cells, amino acid deprivation induces the upregulation of ATF4 mRNA via the PI3K-AKT-NRF2 axis. Simultaneously, activation of the GCN2-p-eIF2α pathway further enhances ATF4 translation, leading to the upregulation of target genes such as asparagine synthase (ASNS), serine, and cystine. Similarly, Chavdoula et al. [46] proposed that the breast cancer oncogene KDM2B circumvents the requirement for eIF2α phosphorylation by directly binding to the ATF4 promoter. Working in concert with MYC, KDM2B transcriptionally regulates ATF4, initiating an adaptive response to amino acid metabolic processes.In colorectal cancer (CRC), Tabata et al. [47] identified the oncogenic factor L2HG as being significantly elevated in tumor tissues. L2HG balances cellular demands for amino acid supply and protein synthesis by activating the mTOR-ATF4 signaling pathway. Collectively, these findings underscore the pivotal role of ATF4 in maintaining amino acid homeostasis within the tumor microenvironment. The metabolic regulation of ATF4 on Gln will be discussed in detail in subsequent sections.

### Induction of tumor angiogenesis

Tumor tissues establish new blood vessels through a “sprouting” process, utilizing the pre-existing microvascular network to meet the demands of tumor growth and metastasis. In the hypoxic environment of the tumor core, the hypoxia-inducible factor-1 (HIF-1) dimeric protein complex is stabilized. Upon activation, ATF4 expression is upregulated, enabling ATF4 to bind to the promoter region of the vascular endothelial growth factor (VEGF) gene, thereby activating VEGF transcription and promoting its secretion [48]. VEGF, a key angiogenic factor, facilitates the proliferation, migration, and lumen formation of tumor vascular endothelial cells [49]. Nutrient deficiency often accompanies hypoxia during tumor ischemia, further driving angiogenesis. Alban et al. [50] demonstrated that sulfur amino acid restriction acts as a pro-angiogenic trigger independent of hypoxia or HIF-1α. Through the GCN2-ATF4 amino acid starvation response pathway, VEGF expression is upregulated in endothelial cells, increasing capillary density in tumor tissues. In addition, glucose deprivation has been shown to significantly elevate VEGF production without involving hypoxia or HIF-1α in initiating the angiogenic switch. Studies on prostate cancer [51] and ovarian cancer [52] confirm this phenomenon. Wang et al. [53] further elucidated this observation by showing that glucose deprivation induces endoplasmic reticulum (ER) stress, which activates the unfolded protein response (UPR) signaling pathway. Specifically, the PERK-eIF2α-ATF4 axis upregulates VEGF expression while simultaneously suppressing the levels of CXCL14, an anti-angiogenic mediator. This creates a pro-angiogenic environment, enhancing tumor blood supply, restoring ER homeostasis, and alleviating environmental stress.

Beyond regulation by tumor cells, tumor angiogenesis is significantly influenced by stromal cells within the tumor microenvironment. A strong correlation has been observed [54] between the number of tumor-associated macrophages (TAMs) and changes in microvascular density. TAMs are recruited and differentiated from macrophages by cytokines and chemokines in the tumor microenvironment. Liu et al. [55], using real-time PCR in a breast cancer mouse model, identified macrophage colony-stimulating factor (M-CSF) as a key factor positively correlated with ATF4 expression. Their findings demonstrated that ATF4 overexpression enhances M-CSF secretion, promoting macrophage infiltration into tumor tissues. This indirectly increases tumor microvascular density and accelerates tumor growth. Interestingly, the mRNA levels of most conventional pro-angiogenic factors, including VEGF-A, PlGF, PDGF-B, Ang-1, and Ang-2, were not significantly elevated, with some even showing a tendency to decrease. These findings suggest that ATF4 may regulate tumor angiogenesis through non-traditional mechanisms.TAMs also contribute to angiogenesis by producing matrix metalloproteinases (MMPs), which degrade the extracellular matrix to create pathways for vascular endothelial cell migration [56]. Zhu et al. [57] reported that in esophageal squamous cell carcinoma, hypoxia activates the unfolded protein response (UPR) signaling pathway via the PERK-eIF2α-ATF4 axis, leading to upregulation of MMP-7 expression. MMP-7 degrades soluble VEGFR-1, preventing VEGF sequestration and thereby increasing VEGF bioavailability. This enhanced VEGF activity promotes angiogenesis while facilitating cancer cell invasion and metastasis.

### Resistance to oxidative stress

Tumor cells frequently encounter elevated oxidative stress due to metabolic reprogramming, such as the Warburg effect, which is characterized by increased glucose uptake and metabolic processes. This reprogramming leads to enhanced electron leakage from complexes I and III of the mitochondrial electron transport chain. The leaked electrons react with oxygen to form superoxide anions (O_2_^−^), initiating a cascade of redox reactions that generate reactive oxygen species (ROS) and exacerbate oxidative stress [58]. Oxidative stress disrupts protein folding within the endoplasmic reticulum, resulting in the accumulation of unfolded proteins. This activates the unfolded protein response (UPR), which engages the PERK-eIF2α-ATF4 pathway to bolster cellular defenses against oxidative stress [59]. In parallel, oxidative stress-induced DNA damage activates poly(ADP-ribose) polymerase-1 (PARP-1), which modifies ATF4 via poly(ADP-ribosyl)ation (PARylation). This modification reduces ATF4’s binding affinity to CRE sequences, inhibiting the expression of MKP-1, preventing MAP kinase inactivation, and exacerbating mitochondrial damage and ROS production [60].

Glutathione (GSH), a vital scavenger of ROS, plays a central role in mitigating oxidative stress. Bai et al. [61] demonstrated in triple-negative breast cancer cells that eIF2α phosphorylation promotes ATF4 activation, enabling it to transcriptionally regulate the glutamate-cysteine ligase catalytic subunit (GCLC) and SLC7A11, which are involved in the reverse transport of cystine and glutamate. ATF4 binds to AP-1 and amino acid response elements in the promoter regions of these genes. Additionally, ATF4 regulates cystathionine gamma-lyase (CTH), responsible for cysteine synthesis, by binding to cis-regulatory intron elements. Collectively, these mechanisms enhance intracellular GSH levels, strengthening ROS scavenging capacity. Torrence [62] further proposed that ATF4 promotes GSH synthesis in response to mTORC1 signaling, specifically by regulating SLC7A11 [63]. In addition to ATF4’s direct regulation, mitochondrial one-carbon metabolic processes also contributes to GSH synthesis. Serine is converted into glycine via the folate-dependent enzyme serine hydroxymethyltransferase 2 (SHMT2), providing a key substrate for GSH biosynthesis. Mijn et al. [64] confirmed that ATF4 knockout in renal cell carcinoma cells slows cysteinyl-glycine production and accelerates GSH degradation. Quirós et al. [65] further elucidated that ATF4 targets key one-carbon metabolic processes enzymes, Aldh1l2 and Mthfd2, during mitochondrial stress. These enzymes facilitate the serine-to-glycine conversion, supporting GSH biosynthesis and mitigating oxidative damage. NRF2, another critical regulator of oxidative stress, activates downstream antioxidant genes, including GSH, thioredoxin (TXN) pathway components, and NADPH regeneration, by binding to antioxidant response elements (AREs). Sarcinelli et al. [66] proposed that PERK-mediated endoplasmic reticulum stress increases ATF4-dependent NRF2 mRNA levels. Alternatively, ATF4 directly regulates NRF2 by binding to CARE sequences in its promoter. Interestingly, ATF4 also functions as a transcriptional activator of cysteinyl-tRNA synthetase 1 (CARS1), which independently promotes NRF2 activation.

### Promotion of apoptosis

The duration and severity of endoplasmic reticulum (ER) stress are key determinants of the shift in the unfolded protein response (UPR) from promoting cell survival to triggering apoptosis. Initially, the UPR aims to restore ER homeostasis. However, under prolonged or severe stress conditions, its pro-apoptotic branch becomes activated. Sequential phosphorylation of eIF2α induces ATF4 expression, which subsequently upregulates CHOP [67]. CHOP facilitates apoptosis by disrupting mitochondrial integrity and amplifying pro-apoptotic signaling [68]. McCullough et al. [69] showed that increased CHOP expression downregulates anti-apoptotic Bcl-2 family proteins, such as BCL-XL and BCL-W, while upregulating pro-apoptotic BH3-only proteins, including BID, BIM, and BAD. CHOP also promotes the formation of BAX-BAK oligomers, which permeabilize the mitochondrial membrane, leading to the release of cytochrome c (Cyt-C). Cyt-C release activates caspase-9, which subsequently activates executioner caspases, including caspase-3 and caspase-7, initiating the apoptotic cascade. In addition to its role in mitochondrial pathways, CHOP upregulates the pseudokinase TRIB3 via an overlap between CHOP binding sites and the amino acid response element in the TRIB3 promoter [70]. Overexpression of TRIB3 enhances phosphorylation of protein kinase B (AKT), which directly regulates the expression of caspase-3, caspase-9, and pro-apoptotic mitochondrial factors such as BAX and BAD [71]. Interestingly, recent studies by Wu et al. [72] revealed that the PERK/ATF4/CHOP pathway induces the transcription of the long non-coding RNA (lncRNA) GOLGA2P10. GOLGA2P10 increases BCL-XL protein levels and promotes BAD phosphorylation, thereby conferring resistance to ER stress-induced apoptosis in tumor cells. These findings underscore the dual role of the PERK/ATF4/CHOP axis, which can either drive apoptosis or promote survival, depending on the downstream regulatory context.

Unexpectedly, Han et al. [73] demonstrated that the forced expression of CHOP alone does not significantly reduce cell viability, while forced expression of ATF4 alone induces apoptosis in cancer cell lines. Surprisingly, CHOP knockdown increased cell death, suggesting that CHOP functions as a secondary signal for ER stress-induced apoptosis, whereas ATF4 plays a more critical role. Han et al. further proposed that ATF4- and CHOP-mediated apoptosis is primarily driven by increased protein synthesis. Both ATF4 and CHOP directly upregulate GADD34, which encodes the regulatory subunit of protein phosphatase 1 (PP1). GADD34 facilitates eIF2α dephosphorylation, enhancing protein translation during ER stress. This surge in protein synthesis generates reactive oxygen species (ROS) and depletes ATP, ultimately driving apoptosis.

## Glutamine metabolism in tumors

Gln serves as a “conditionally essential” amino acid with a pivotal role in tumor cell metabolism. Although one of the most abundant free amino acids in the body, the rapid cancer cell proliferation often creates a demand exceeding endogenous production and exogenous supply, leading to localized Gln depletion within tumor tissues. Gln provides nitrogen and carbon sources essential for tumor growth and exhibits two key characteristics: “Gln addiction” and a vital role in fueling the tricarboxylic acid (TCA) cycle [74]. Tumor expansion further intensifies Gln scarcity in poorly vascularized areas due to preferential uptake by cancer cells, leading to spatial heterogeneity [75]. Advanced imaging modalities, such as 18F-FDG PET, enable in vivo visualization of Gln metabolism and facilitate tumor monitoring [76].

Tumor cells depend heavily on enhanced Gln metabolism to meet their energy, biosynthetic, and redox demands under rapid proliferation and stress. This dependency, termed “Gln addiction,” underscores Gln’s role in supporting tumor survival and metastasis [77]. Gln is synthesized from glutamate and ammonia by glutamine synthetase (GS/GLUL), then imported via transporters such as ASCT2/SLC1A5 and LAT1 [78]. Mitochondrial glutaminase (GLS/GLS2) deaminates Gln to glutamate, which is further metabolized through two principal pathways: (1) conversion to α-ketoglutarate (α-KG) by glutamate dehydrogenase (GDH) or aminotransferases; (2) synthesis of glutathione (GSH) via γ-glutamate-cysteine ligase (GCL) and glutathione synthase (GSS), enhancing antioxidant defenses [79, 80].

**Fig. 2:**
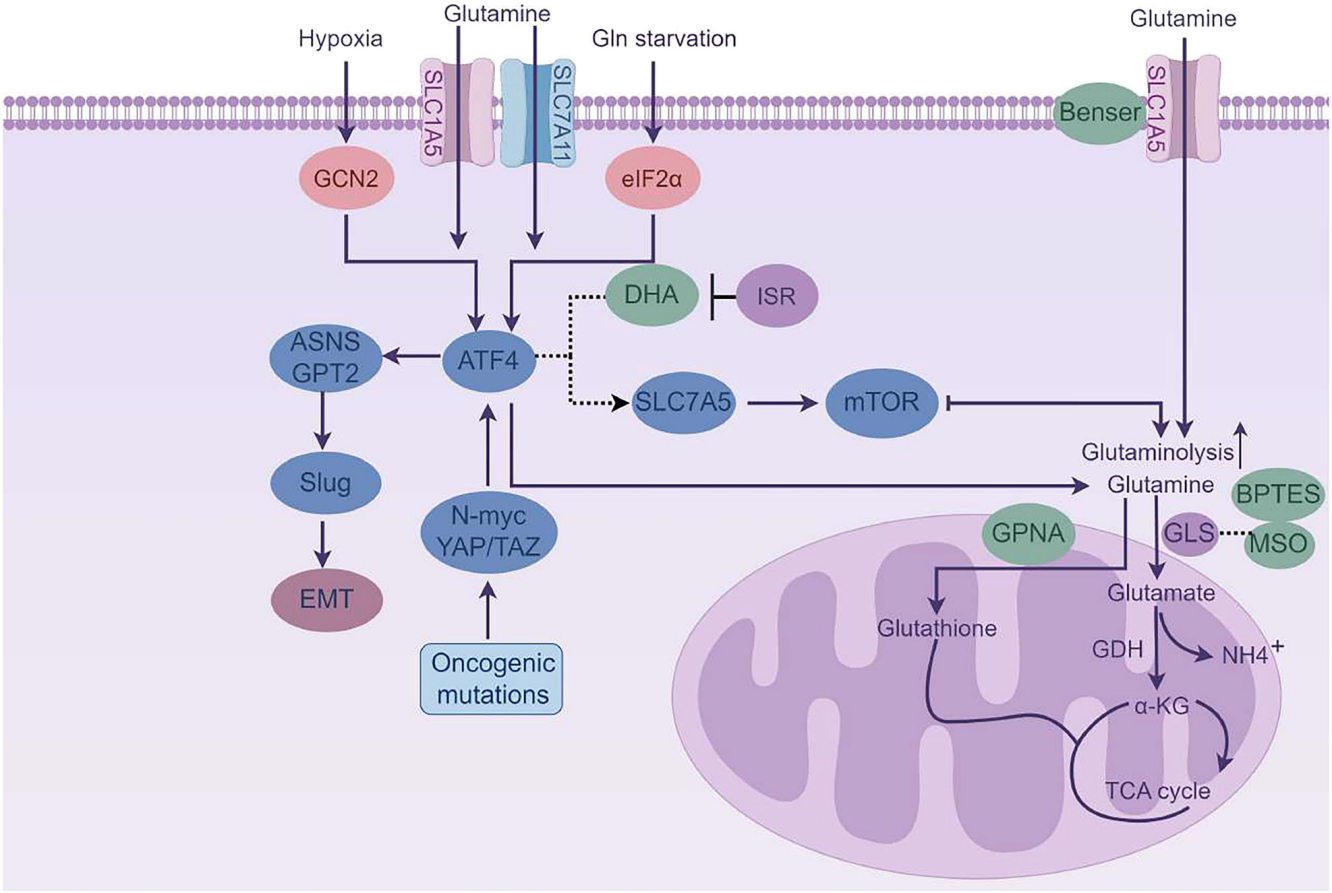
Glutamine metabolism reprogramming.

Moreover, Gln-derived nitrogen is critical for nucleotide biosynthesis, contributing directly to purine and pyrimidine synthesis required for DNA and RNA production, thereby supporting cell cycle progression (Fig. 2). Tumor cells exhibit a distinct metabolic phenotype: despite adequate oxygen, they preferentially metabolize glucose to lactate through glycolysis rather than oxidative phosphorylation, a phenomenon initially termed the Warburg effect [78, 81]. Subsequent studies revealed that many tumors retain functional mitochondria [82] but divert glucose carbons away from the TCA cycle.

Instead, tumors rely on Gln anaplerosis to replenish TCA cycle intermediates. Under normoxia, α-KG derived from Gln feeds the canonical TCA cycle; under hypoxia or mitochondrial impairment, α-KG undergoes reductive carboxylation to citrate [83, 84]. Cytoplasmic citrate is cleaved by ATP citrate lyase to acetyl-CoA, which serves as a substrate for fatty acid synthesis supporting membrane biosynthesis and growth [44]. Thus, Gln metabolism complements glycolytic flux to sustain tumor bioenergetics and biosynthesis.

Pharmacological inhibition of Gln metabolism attenuates glycolytic lactate production and disrupts tumor energy homeostasis [85]. Immune evasion, a key hallmark of cancer, is intricately linked to Gln metabolism. Immune cells within the nutrient-limited tumor microenvironment rely on Gln uptake and catabolism for activation and effector functions [86–88]. Activated T effector cells upregulate glycolysis and Gln metabolism, with increased expression of transporters like SLC1A5 and SLC38A1 [89].

GLS1 deletion shifts T cell differentiation, favoring Th1 and CD8^+^ cells over Th17 via modulation of T-bet and mTORC1 signaling, thereby impacting immune responses [90–92]. Conversely, Gln deprivation promotes regulatory T cell (Treg) induction via AMPK-mTORC1 pathways, suppressing effector T cell function [93]. Competition for Gln between tumor and immune cells creates a metabolic tug-of-war that impairs antitumor immunity.

Therapeutic targeting of tumor Gln metabolism can restore amino acid availability to immune cells and enhance antitumor responses. For instance, Hongbo Chi et al. [94] demonstrated that tumor and dendritic cells compete for Gln via SLC38A2, and Gln supplementation rescues dendritic cell function and CD8^+^ T cell activation, overcoming immunotherapy resistance. Gln also supports B cell differentiation and antibody production, which decline upon inhibition of Gln transporters or GLS [95, 96].

Additionally, Gln deprivation induces PD-L1 expression in tumor cells, facilitating immune escape. In bladder cancer, Gln starvation activates EGFR/MEK/ERK/c-Jun signaling to upregulate PD-L1 [97, 98], while lung tumors show increased PD-L1 associated with GSH depletion [99]. Restoration of Gln levels reverses PD-L1 expression, and blocking PD-1/PD-L1 interaction diminishes immune evasion, offering therapeutic potential. In summary, Gln metabolism plays a multifaceted role in tumor progression and immune modulation. Targeting this axis offers promising strategies to inhibit tumor growth and potentiate antitumor immunity.

## The ATF4-glutamine metabolic axis in the tumor environment

ATF4, a stress-responsive transcription factor, orchestrates adaptive metabolic responses under cellular stress. Environmental factors reciprocally regulate ATF4 expression, forming a feedback regulatory loop that enables tumor cells to survive and proliferate under metabolic stress, nutrient deprivation, and oxidative conditions.

Key enzymes involved in Gln synthesis and transport are significantly overexpressed in various cancers [100, 101]. Tumor cells enhance plasma Gln uptake via specific amino acid transporters to satisfy metabolic demands. Patel et al. [102] reported a positive correlation between ATF4 expression and elevated Gln transporter (SLC1A5, SLC7A11) levels in estrogen receptor-positive breast cancer, associated with poor prognosis. Xiao et al. [103] identified that coptisine stabilizes the ATF4 promoter G-quadruplex, suppressing the GCN2-ATF4-SLC1A5 axis under Gln restriction in NSCLC, thereby impairing Gln uptake and tumor growth. Ren et al. [104] demonstrated that ATF4 cooperates with N-Myc to transcriptionally activate SLC1A5, increasing Gln dependency in MYCN-amplified neuroblastoma. Upon GLS inhibition by CB-839 in triple-negative breast cancer, ATF4 accumulates, triggering the integrated stress response (ISR), upregulating genes related to amino acid biosynthesis and transport (e.g., PYCR1, ASNS, SLC7A5), and suppressing mTOR signaling [105].

ATF4, as a central regulator of cell metabolic processes, modulating gene expression according to nutrient availability. Albert et al. [106] showed that oncogenic mutations in lung adenocarcinoma activate the ISR and ATF4, inducing asparagine synthetase (ASNS) to promote asparagine synthesis from aspartate and Gln. Kiesel et al. [107] found that hypoxia-activated ATF4 induces glutamate pyruvate transaminase (Gpt2) in metastatic breast cancer, modulating glutamate levels and suppressing Gln catabolism.

Tumor microenvironmental stresses, including hypoxia and nutrient limitation, trigger Gln starvation-induced endoplasmic reticulum stress, leading to GCN2-mediated phosphorylation of eIF2α and upregulation of ATF4. Elevated Gln metabolism is a hallmark of malignancy. Maria et al. [108] found that Gln deprivation activates MEK/ERK signaling, increasing ATF4 and Slug expression, thereby promoting epithelial-mesenchymal transition (EMT) and metastasis in pancreatic cancer.

The Hippo pathway integrates cellular nutrient status and mechanical signals. Kim et al. [109] demonstrated that YAP/TAZ co-activators directly regulate SLC1A5 and SLC38A1 transcription, modulating Gln uptake and glutamate production. YAP/TAZ depletion impairs tumor Gln metabolism and growth. ATF4 is a direct transcriptional target of YAP/TAZ, linking mechanotransduction to metabolic adaptation. Gln metabolism also regulates redox homeostasis. Zhang et al. [110] showed that ATF4 mediates amino acid response (AAR) pathways to sustain cysteine uptake and glutathione synthesis, protecting cells from ferroptosis. Furthermore, ATF4 influences immune evasion through PD-L1 regulation.

In conclusion, the ATF4-Gln metabolic axis plays a crucial role in tumor adaptation to metabolic stress, supporting survival, proliferation, and immune modulation. Targeting this axis holds therapeutic promise to disrupt tumor metabolism and enhance anticancer treatments.

## Potential therapeutic strategies targeting the ATF4-glutamine metabolic axis

The ATF4-Gln metabolic processes axis is increasingly recognized as a key participant in cancer cell metabolic reprogramming. Its role spans multiple aspects of tumor biology, providing an essential foundation for cancer cell survival, proliferation, and resistance to therapy. However, achieving effective clinical translation requires overcoming numerous challenges related to tumor heterogeneity, metabolic plasticity, and potential toxicity. With continued research and the integration of precision medicine approaches, targeting the ATF4-Gln axis holds significant potential to transform cancer treatment paradigms and improve patient outcomes.

Currently, strategies involving artificial semi-synthetic derivatives (compounds) to specifically enhance ATF4 degradation or inhibit its transcriptional activity are being actively explored. Dihydroartemisinin (DHA), the primary active metabolite with antimalarial efficacy, has been shown by Ji et al. [111] to induce cytoplasmic translocation and degradation of ATF4. This downregulates the transcriptional expression of SLC7A11 (also known as xCT, the cystine/glutamate antiporter), promoting lipid peroxidation and inducing ferroptosis in hepatocellular carcinoma cells. Targeting the ATF4-xCT-GSH axis is now regarded as a primary approach to overcoming ferroptosis resistance. Zhang et al. [112] further identified that the small-molecule cyclin-dependent kinase (CDK) inhibitor Hesperadin increases the expression of growth arrest and DNA damage-inducible protein 45α (GADD45A) in pancreatic cancer cells via ATF4 upregulation. This leads to mitochondrial damage, reactive oxygen species (ROS) generation, and apoptosis. Combining radiation therapy with anticancer drugs often causes collateral damage to normal cells due to toxicity. Kim et al. [113] found that the natural compound Fisetin, when used in conjunction with radiation therapy, releases the endoplasmic reticulum (ER) stress-related protein GRP78 into the extracellular space via exosomes in cancer cells. This activates the PERK-ATF4-CHOP pathway and inhibits epithelial-mesenchymal transition (EMT) in radiation-resistant liver cancer cells, thereby overcoming radioresistance. Similarly, Feng et al. [114] proposed that restoring ATF4 expression by inhibiting proteasomal degradation may be a potential strategy to reverse chemoresistance in gastric cancer. Their research demonstrated that after cisplatin treatment, activated CK1δ phosphorylates ATF4 at serine 219, triggering βTrCP-mediated ubiquitination and subsequent proteasomal degradation. The inhibitor bortezomib (BTZ) restores ATF4 protein levels and effectively overcomes chemoresistance by enhancing gastric cancer cell apoptosis. Interestingly, metformin, a widely known drug for diabetes management, primarily exerts its effects by inhibiting mitochondrial respiratory complex I and activating AMP-activated protein kinase (AMPK) to counteract energy depletion [115]. However, Jang et al. [116] discovered that metformin independently inhibits mTORC1 signaling, significantly reducing the viability of non-small cell lung cancer (NSCLC) cells. This effect is contingent on the upregulation of ATF4-induced REDD1 and Sestrin2 expression.

Blocking key pathways of Gln metabolic processes has become a crucial target in tumor metabolic regulation, particularly by inhibiting transporters and key enzymes involved in Gln synthesis and catabolism. Cetuximab resistance is a major obstacle in the treatment of metastatic colorectal cancer (mCRC) [117]. SLC1A5, the primary transporter responsible for Gln uptake into rapidly proliferating cells (including cancer cells), is a promising target. Ma et al. [118] demonstrated that inhibiting SLC1A5 promotes EGFR degradation through the ubiquitin-proteasome pathway and reduces nuclear EGFR expression, thereby improving cetuximab resistance. Similarly, Ye et al. [119] proposed that SLC1A5 inhibitors (e.g., BenSer) and Gln synthetase (GS) inhibitors (e.g., MSO) both significantly reduce intracellular glutamate (Glu) levels in gastric cancer cells. More importantly, their combined treatment has superior effects in inhibiting cancer cell growth and proliferation compared to either inhibitor alone. Small-molecule inhibitors, such as Benzylserine, l-γ-Glutamyl-p-nitroanilide (GPNA), V-9302, and γ-FBP, are widely recognized for their ability to selectively and effectively target SLC1A5, subsequently reducing glutathione (GSH) synthesis. These metabolic changes increase cancer cells’ sensitivity to ROS-induced apoptosis [120]. Laboratories are also leveraging integrated experimental and computational methods to rationally design compound inhibitors of SLC1A5, such as Lc-BPE [121] and γ-FBP [122]. Monoclonal antibodies have been shown to possess higher specificity and stability compared to traditional small-molecule inhibitors. Suzuki et al. [123] developed monoclonal antibodies (e.g., KM4008, KM4012, KM4018) using hamster ovary cells expressing SLC1A5 as immunogens. These antibodies successfully recognized SLC1A5 surface domains and inhibited the proliferation of Gln-dependent cancer cells. The intracellular processing of Gln begins with its conversion to glutamate catalyzed by glutaminase (GLS), which is also a potential therapeutic target. The first GLS inhibitor applied in preclinical models, 6-diazo-5-oxo-l-norleucine, has largely been abandoned due to its non-specificity and adverse effects. However, a small-molecule inhibitor, BPTES, capable of simultaneously inhibiting GLS and GLS2, has been developed. BPTES has demonstrated efficacy in glioma [124], breast cancer [125], and other tumor models by reducing glutamate and α-ketoglutarate (α-KG) levels, subsequently decreasing TCA cycle intermediates and their downstream products, thereby slowing tumor growth. Low-dose gemcitabine induces cell senescence and promotes treatment resistance in pancreatic ductal adenocarcinoma through EMT induction. GLS1 expression is elevated in senescent cells. Oyama et al. [126] found that combining BPTES with gemcitabine enhances chemotherapy efficacy by inhibiting the proliferation and inducing apoptosis of senescent cancer cells without causing resistance. Today, the development of BPTES derivatives, such as CB-839, has gained significant traction. Nanoparticles containing BPTES and chemotherapeutic drugs are also being tested in vitro. Beyond developing inhibitors, researchers are employing strategies such as targeted degradation, prodrugs, radiopharmaceuticals, and boron neutron capture therapy to address the overexpression of Gln transporters and key enzymes in tumor cells. These approaches further expand the potential applications of targeting Gln metabolic processes pathways and show considerable promise for clinical translation.

## Conclusion

The interaction between ATF4 and Gln metabolic processes underscores the complexity and adaptability of tumor metabolic reprogramming. As a key regulator of the integrated stress response (ISR), ATF4 equips cancer cells with enhanced survival and adaptation capabilities by modulating Gln metabolic processes and the expression of related genes in response to nutrient and oxidative stress. This axis not only supplies energy and metabolic intermediates for tumor cells but also drives tumor growth and metastasis by promoting immune evasion, oxidative stress tolerance, and angiogenesis. Consequently, the ATF4-Gln metabolic axis represents a critical target for understanding the mechanisms underlying cancer initiation and progression.

Despite the promising prospects of therapeutic strategies targeting the ATF4-Gln metabolic axis, several challenges remain. Tumor heterogeneity and metabolic flexibility contribute to significant variations in treatment efficacy, while drug toxicity and potential adverse effects on normal cell metabolic processes require further optimization. Future advancements in artificial intelligence and multi-omics technologies offer an opportunity to identify more precise targeting strategies and develop safer, more effective therapeutic agents. Additionally, combining inhibitors of this axis with traditional chemotherapy or immunotherapy may further enhance treatment outcomes.

In conclusion, the central role of the ATF4-Gln metabolic axis in tumor metabolic processes provides a pivotal target for precision oncology. Future research should focus on unraveling its intricate regulatory mechanisms and refining targeted therapeutic regimens to achieve more effective cancer treatments and improved patient outcomes.
